# Supplementary material for: Oroxylum indicum (L.) Leaf Extract Attenuates β-Amyloid-Induced Neurotoxicity in SH-SY5Y Cells
Source: Int J Mol Sci. 2025 Mar 23;26(7):2917. doi: 10.3390/ijms26072917 (PMC11988460; doi:10.3390/ijms26072917)
Supplement: Supplementary file 1 [file ijms-26-02917-s001.zip › ijms-3550042-supplementary.pdf]

Originate for figure 2A (control)

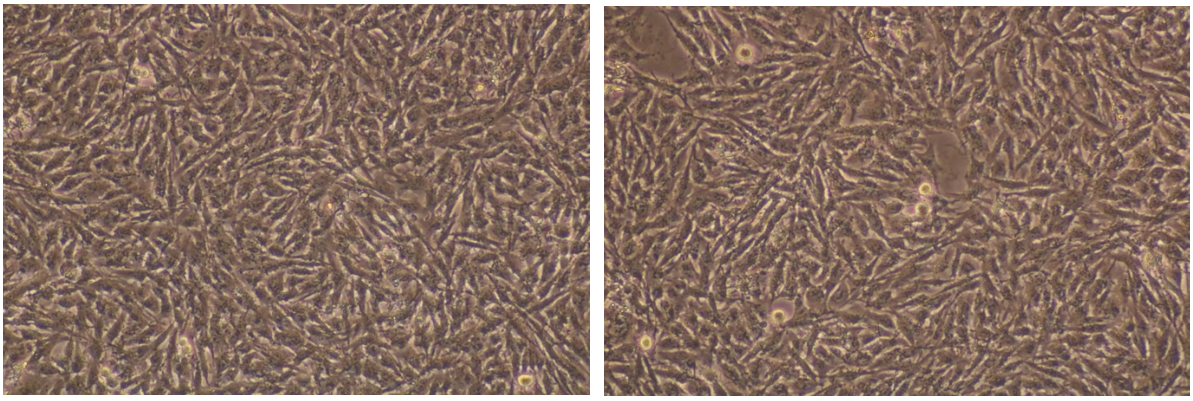

Originate for figure 2A (beta amyloid)

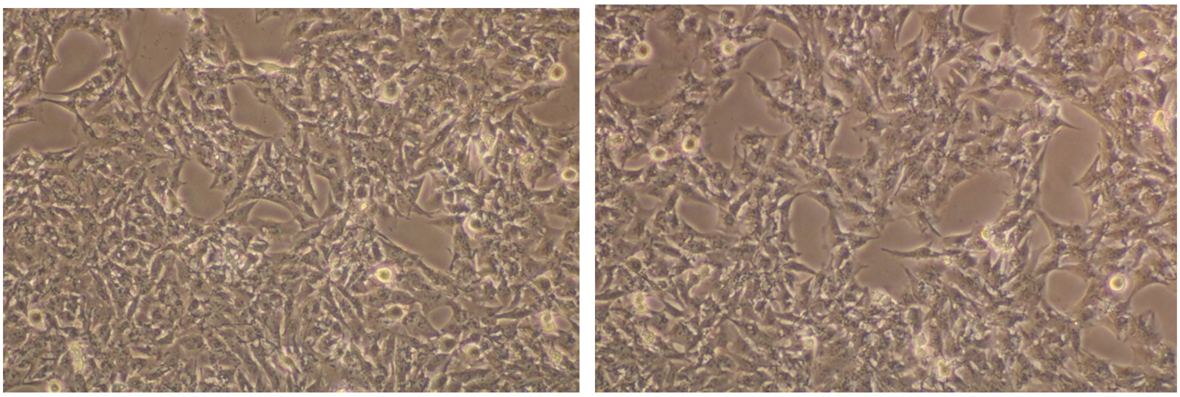

Originate for figure 2A (beta amyloid+OIL 25μg/ml)

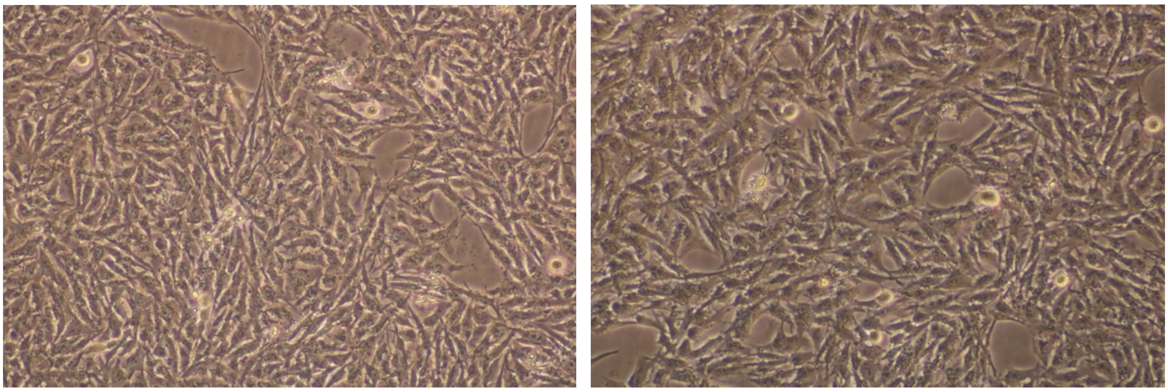

Originate for figure 2A (beta amyloid+OIL 50μg/ml)

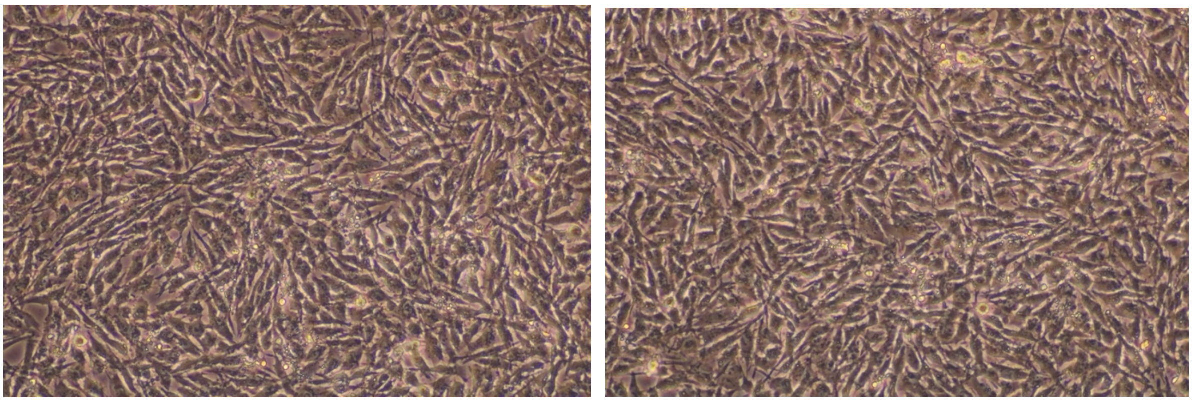

Originate for figure 3B

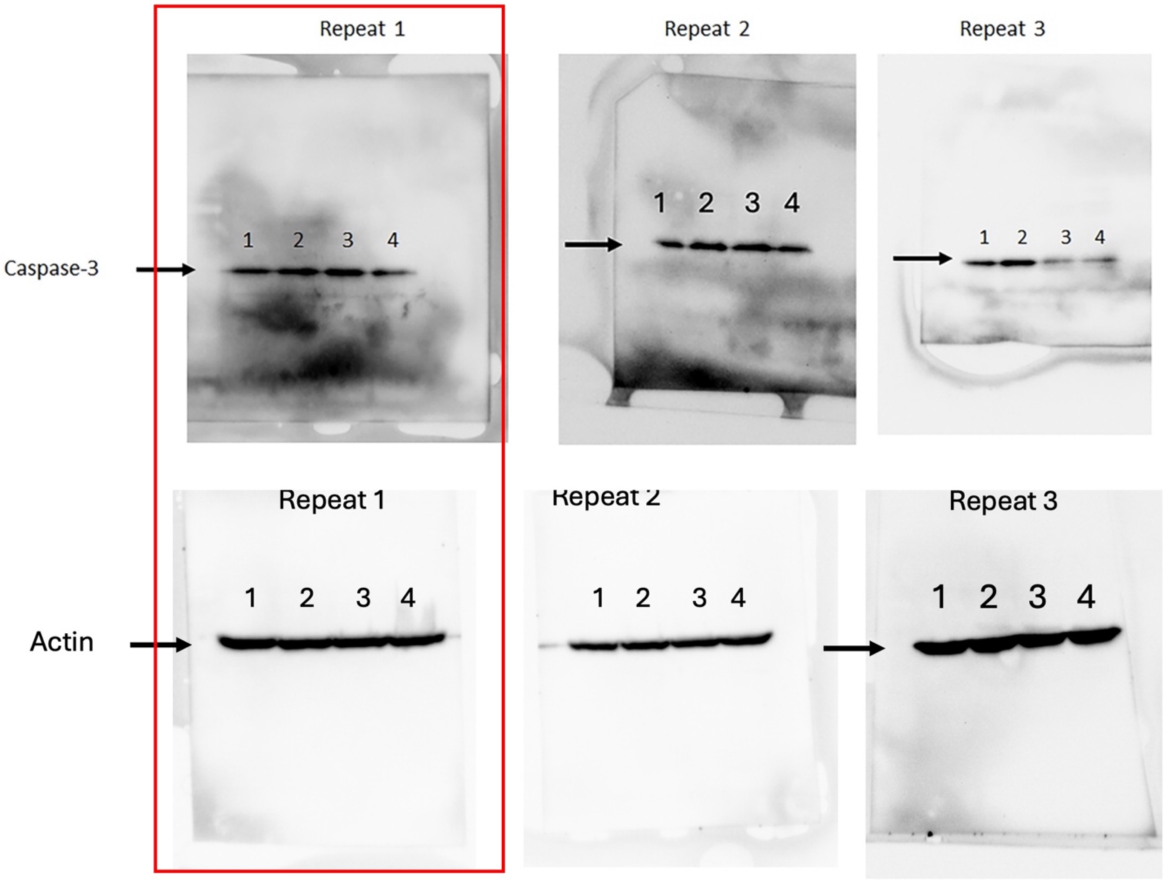

Western blot analysis of caspase-3 expression in SH-SY5Y cells.
The experimental groups included:
(1) control,
(2) Aβ,
(3) Aβ+ OIL (25 μg/mL), and
(4) Aβ+ OIL(50 μg/mL).

Originate for figure 4A

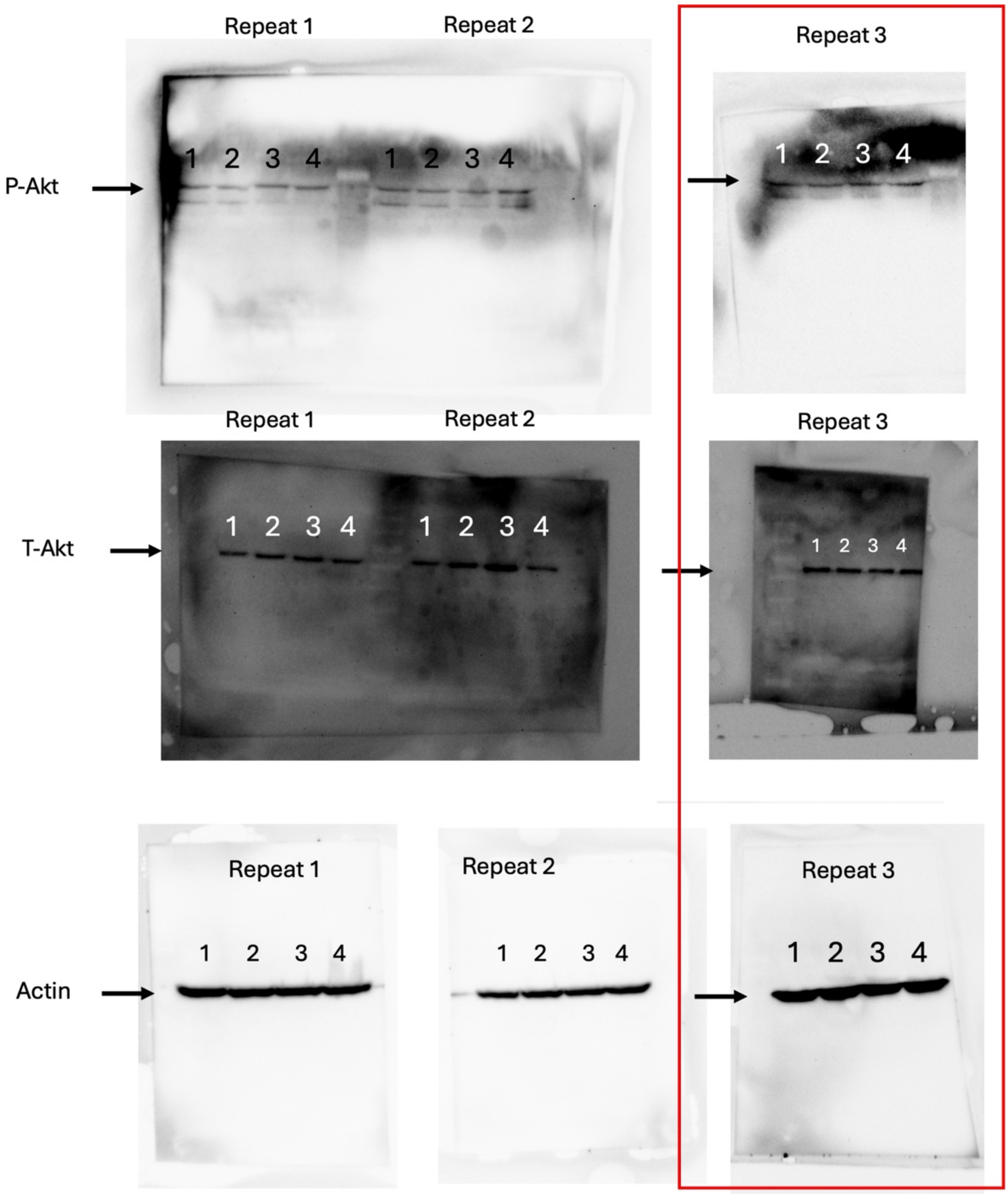

Western blot analysis of p-Akt, T-Akt and beta actin in SH-SY5Y cells.
The experimental groups included:
(1) control,
(2) Aβ,
(3) Aβ+ OIL (25 μg/mL), and
(4) Aβ+ OIL(50 μg/mL).

Originate for figure 4B

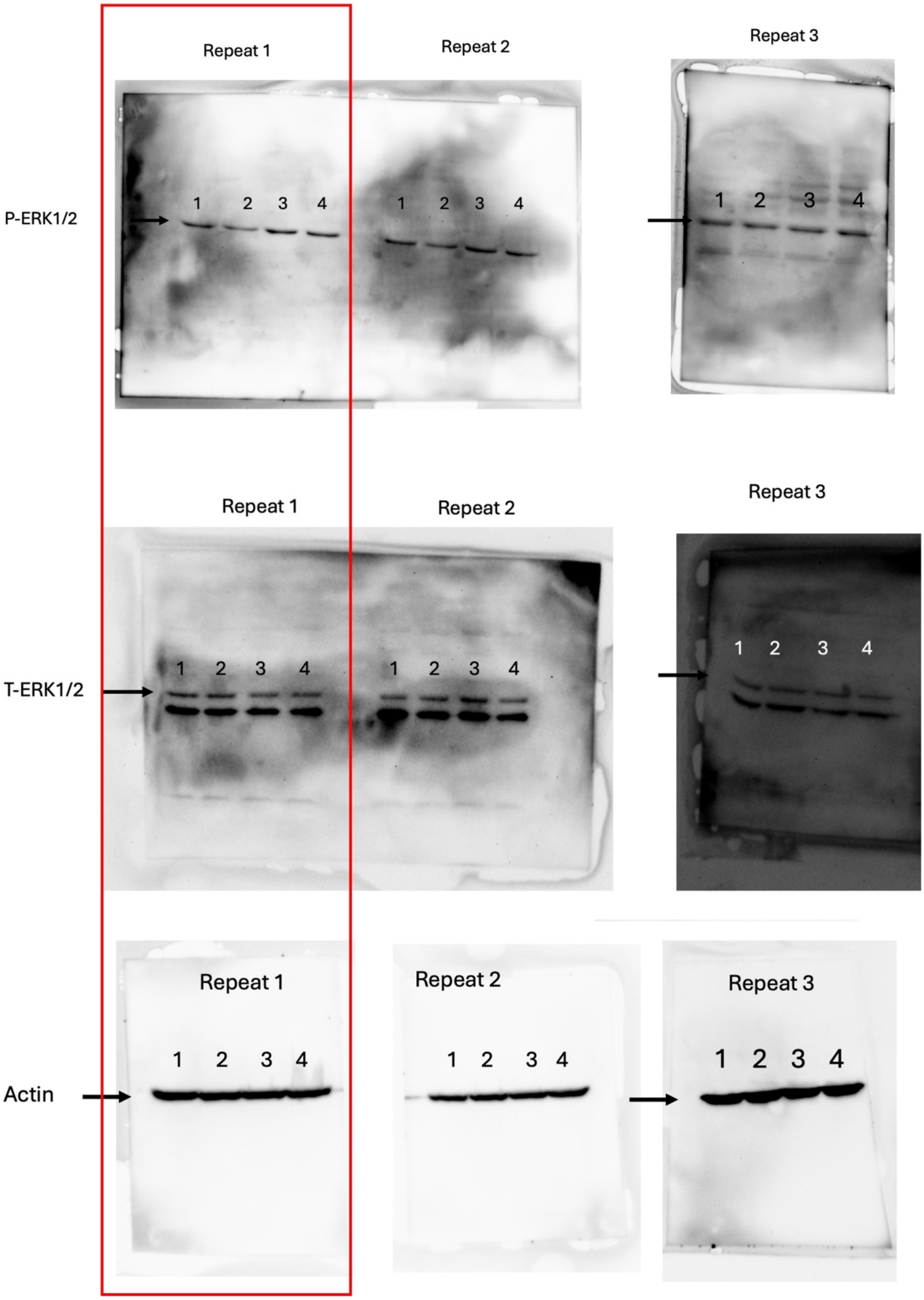

Western blot analysis of p-ERK, T-ERK and beta actin in SH-SY5Y cells.
The experimental groups included:
(1) control,
(2) Aβ,
(3) Aβ+ OIL (25 μg/mL), and
(4) Aβ+ OIL(50 μg/mL).

Originate for figure 5A

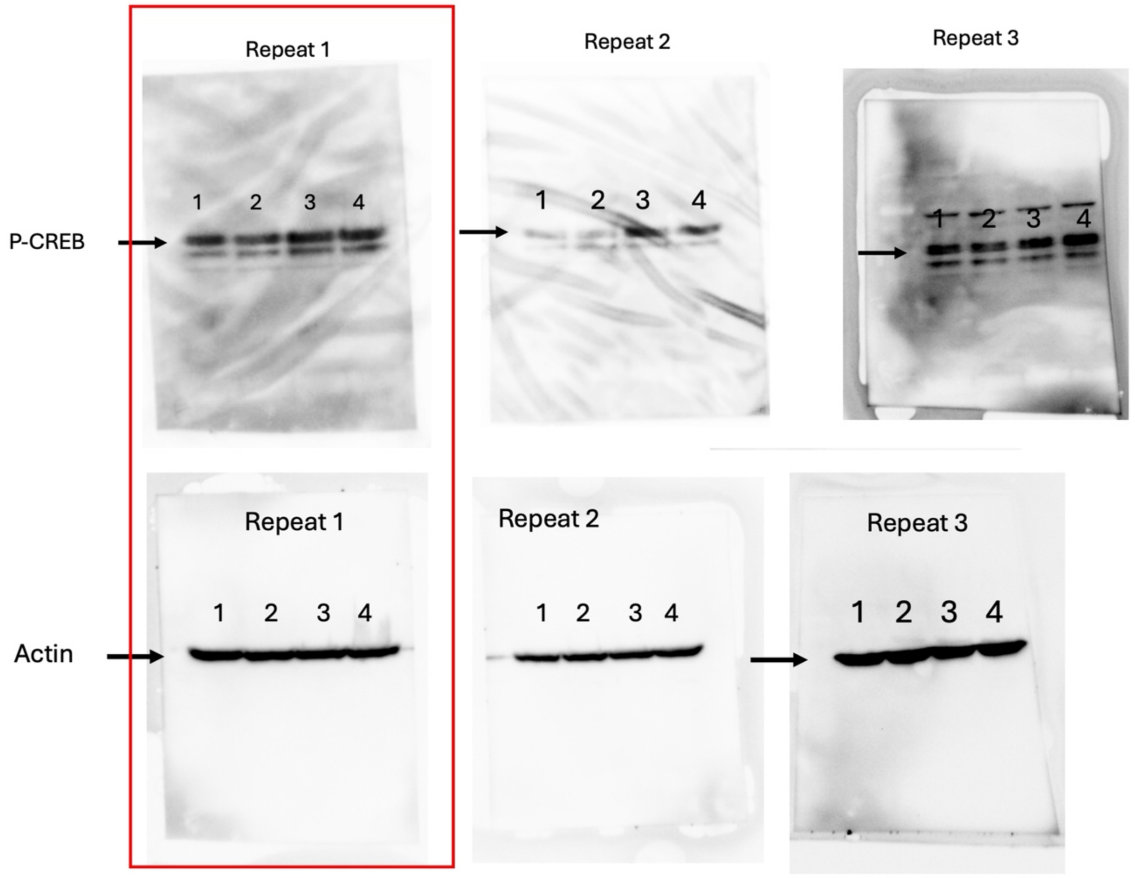

Western blot analysis of P-CREB, and beta actin in SH-SY5Y cells.
The experimental groups included:
(1) control,
(2) Aβ,
(3) Aβ+ OIL (25 μg/mL), and
(4) Aβ+ OIL(50 μg/mL).

Originate for figure 5B

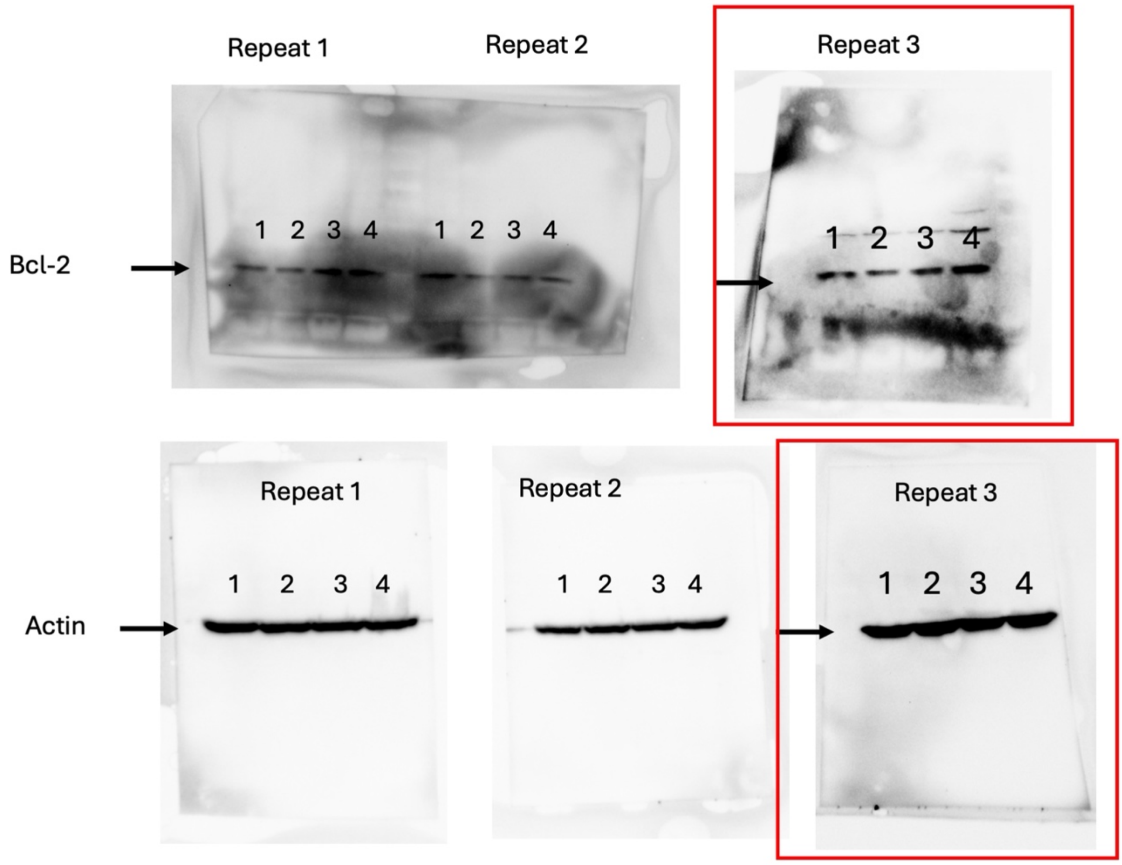

Western blot analysis of Bcl-2, and beta actin in SH-SY5Y cells.
The experimental groups included:
(1) control,
(2) Aβ,
(3) Aβ+ OIL (25 μg/mL), and
(4) Aβ+ OIL(50 μg/mL).

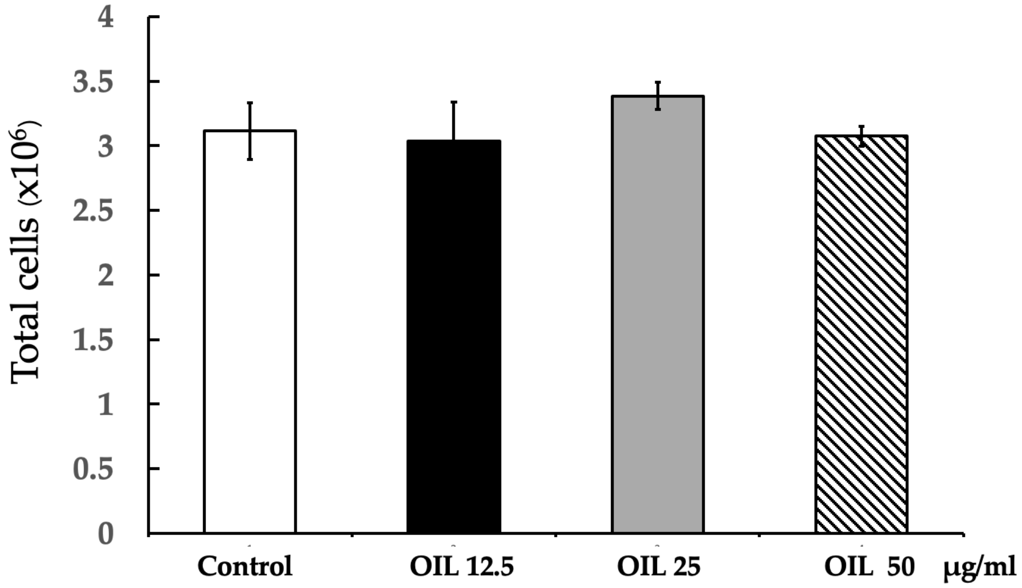

Supplement to Figure 1B. To verify that OIL does not significantly enhance cell proliferation, cell counts were conducted in both the control group and the OIL-treated group at dosages of 12.5, 25, and 50 µg/ml. The results demonstrated that OIL-treated cells did not exhibit a significant increase in cell number compared to the control group.
